# Peritoneal Dialysis Catheter Emplacement by Advanced Laparoscopy: 8-year Experience from a Medical Center of China

**DOI:** 10.1038/s41598-017-09596-1

**Published:** 2017-08-22

**Authors:** Min Mo, Yongle Ju, Haitang Hu, Wei Zhang, Jianyi Pan, Qingkun Zheng, Jinzhong Chen, Lijuan Su, Xianrui Dou

**Affiliations:** 1 Department of Nephrology, Shunde Hospital of Southern Medical University, Shunde, 528300 China; 2 Gastro-entero-pancreatic laparoscopic training centre, Shunde Hospital of Southern Medical University, Shunde, 528300 China

## Abstract

Laparoscopic experience and relevant reports about PD catheter emplacement in Chinese patients are seldom. In this study, we described our experience with advanced laparoscopy for PD catheter implantation in Chinese patients. There were one hundred and thirty Chinese patients accepted advanced laparoscopic approach for PD catheter emplacement in this study. Six of 26 patients with prior abdominal operations had abdominal adhesion, while six of 104 patients without prior abdominal surgeries showed abdominal adhesion. Operation time required 10 to 180 minutes. During a mean follow-up time of 26.46 months, the catheter complications were shown as outflow obstruction (n = 6, 4.62%), pericatheter leaking (n = 3, 2.31%), hydrocele of tunica vaginalis (n = 1, 0.77% in all), and umbilical hernia (n = 2, 1.54%). Cumulative revision-free survival probability for catheter loss from mechanical complications at 8 years was 0.95. During the postoperative follow-up ranged between 6 and 106 months, 98 patients (75.38%) were still on CAPD, 17 patients (13.08%) died, 8 patients (6.15%) were transferred to hemodialysis, 6 patients (4.62%) received kidney transplantation, and 1 patient (0.77%) showed improved renal function. These results showed that PD catheter placement with advanced laparoscopy is a safe and effective approach in Chinese patients with or without prior abdominal surgeries.

## Introduction

Peritoneal dialysis (PD) is one of the most important renal replacement therapies for end-stage renal disease (ESRD) patients. As a home therapy, PD affords better patient autonomy and life quality than in-center hemodialysis^1, 2^. The catheter placement is the first step for PD, and the availability of permanent and functional peritoneal access is the most important factor for the success of long-term PD^3^. Since the early 1990s, laparoscopy has been applied in the insertion of PD catheters. A large number of reports showed that this technique has some advantages over other techniques because of direct vision, being assure of right position of the catheter, and reducing catheter complications^4^. However, the most of literatures about laparoscopic catheter emplacement were Occidental, and the surgical experience was mostly from Occidental patients^5–7^. Reports about laparoscopic PD catheter placement in Chinese patients were seldom with small sample size. Hereby, the purpose of the present study is to introduce our experience in establishing PD access in Chinese patients by employing an advanced laparoscopic approach, as wells as the characteristics of catheter complications and the patient outcomes.

## Results

### Patient characteristics

A total 130 patients (86 males and 44 females) with end stage renal diseases were recruited in this study. The mean age was 46.43 ± 14.04 years old. Among these patients, 26(20%) had previous history of abdominal operations. The causes of end stage renal disease include chronic glomerular nephritis (n = 79), hypertension (n = 22), diabetic nephropathy (n = 12), obstructive nephropathy (n = 8), polycystic kidney disease (n = 4), lupus nephritis (n = 3), and others (n = 2). Patient demographics were listed in Table 1.

**Table 1:** Demographics of Patients.

Number of patients(n)	Total (130)
Age in years, Mean ± SD (Rang)	46.43 ± 14.04 (Range, 15–83)
Gender (n, (%))	
Male	86(66.15)
Female	44(33.85)
Cause of ESRD (n, (%))	
Chronic glomerular nephritis	79(60.77)
Hypertension	22(16.92)
Diabetic nephropathy	12(9.23)
Obstructive nephropathy	8(6.15)
Polycystic kidney disease	4(3.08)
Lupus nephritis	3(2.31)
Others	2(1.54)
Prior abdominal surgery (n, (%))	
with	26(20)
without	104(80)

Note: n, number of observations; SD, standard deviation; ESRD, end-stage renal disease.

### Operative characteristics during PD catheter placement with laparoscopy

Of the 26 attempted catheter insertion procedures in which the patients had a previous history of abdominal operations, 6 (23.08%) patients showed adhesions, while 6 of 104 (5.77%) patients without prior surgery displayed adhesions. After anesthesia, the operation only took about 10 minutes in patients with no need of adhesiolysis or other additional operations. If the adhesiolysis was required during the operation, it took 30 minutes to 3 hours. The successful rate of PD catheter placement without abandoning the procedure was 100%. There were no episodes of bowel/viscera perforation in all patients. One patient with early postoperative bleeding was found the rectus errhysis in the trocar location. Operative characteristics during PD catheter placement with laparoscopy were shown in Table 2.

**Table 2:** Operative characteristics during PD catheter placement with laparoscopy.

Abdominal status	Total (n = 130)
Without prio abdominal surgery (n)	104
Without abdominal adhesion (n, (%))	98(94.23)
Abdominal adhesion (n, (%))	6(5.77)
With prio abdominal surgery(n,)	26
Without abdominal adhesion (n, (%))	20(76.92)
Abdominal adhesion (n, (%))	6(23.08)
Large or bulky omentum (n)	7
Operation time(minutes)	10–180
Don’t need adhesiolysis	10
Need adhesiolysis	30–180
Operative complications	
Failed placement (n, (%))	0(0)
Bowel/viscera perforation (n, (%))	0(0)
Early postoperative bleeding (n, (%))	1(0.77)

Note: n, number of observations.

### Clinical complhications and outcomes

**Figure 1:**
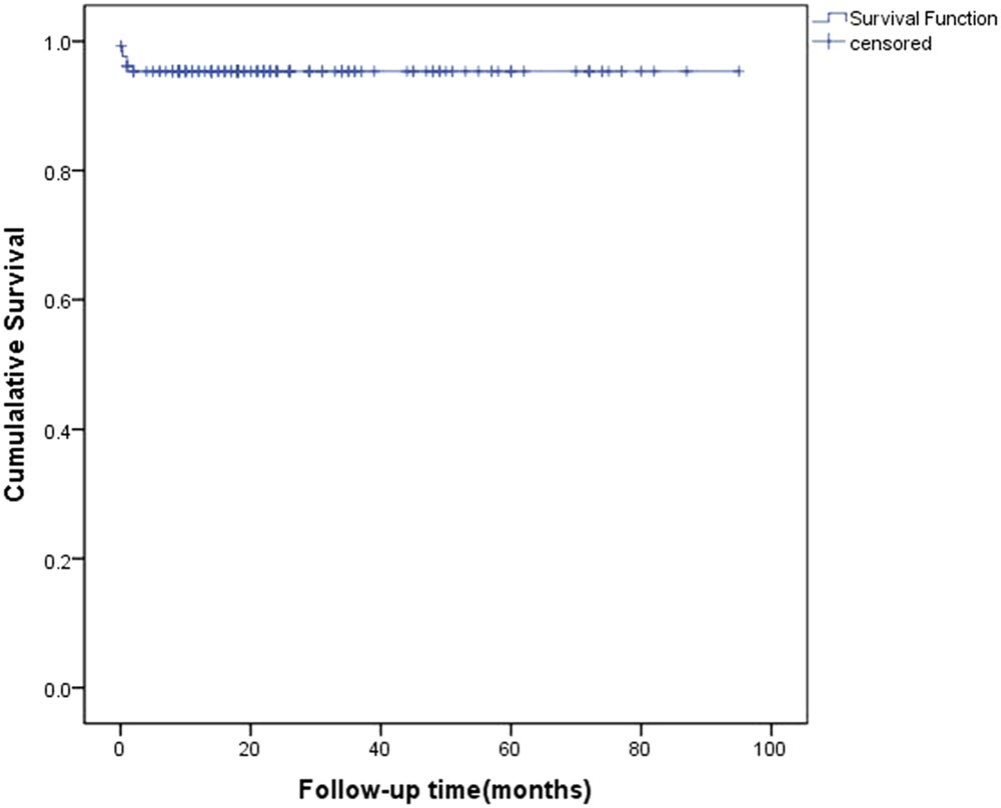
Cumulative revision-free catheter survival probability for mechanical complications. This figure showed the probability of 106-month catheter survival free of revisions for mechanical complications. The revision-free catheter survival probability was 0.95 by the end of 8 years follow-up.

All patients started training of peritoneal dialysis as planned. All patients (130 patients) received the follow-up more than half a year. During the follow-up of first half year, 6 patients (4.62%) occurred outflow obstruction, 3 patients (2.31%) occurred pericatheter leaking, 1 patient (0.77%) occurred hydrocele of tunica vaginalis, and 1 patient (0.77%) developed umbilical hernia complication. The occurrences of catheter outflow obstruction or pericatheter leaking were within three months of postoperation in 130 patients. There were no new occurrences of catheter outflow obstruction or pericatheter leaking after three months of PD catheter placement in all patients, while one patient developed umbilical hernia complication at twelve months of PD. The catheter complications during the follow-up from 3 months to one year are shown in Table 3. During a mean follow-up of 26.46 months, the catheter complications were shown by 4.62% outflow obstruction, 2.31% pericatheter leaking, 0.77% hydrocele of tunica vaginalis, and 1.54% umbilical hernia. The occurrences of catheter outflow obstruction or pericatheter leaking only occurred in the first three months of postoperation of PD catheter placement in all patients. The number of catheter loss due to mechanical complications was 6(4.62%). Revision-free catheter survival probability was 0.95 by the end of 8 years follow-up. The Revision-free catheter survival probability for mechanical complications is shown in Fig. 1. During the follow-up period between 6 and 106 months, 17 patients (13.08%) died (the causes of death: 6 peritonitis, 9 cardiovascular and cerebrovascular disease, 2 other disease), 8 patients (6.15%) converted to hemodialysis (5 peritonitis, 1 omental wrapping, 1 ultrafiltration failure, 1 being unable to perform PD on his own), 6 patients (4.62%) received kidney transplantation, and 1 patient (0.77%) showed improved renal function. 98 patients (75.38%) remained on continuous ambulatory peritoneal dialysis (CAPD).

**Table 3:** The catheter complications during the follow-up from 3 months to 12 months.

Catheter complications	Follow-up period			
	3-months	6-months	9-months	12-months
	n = 130	n = 130	n = 129	n = 119
Outflow obstruction (n, (%))	6(4.62)	6(4.62)	6(4.65)	6(5.04)
Pericatheter leaking (n, (%))	3(2.31)	3(2.31)	3(2.33)	3(2.52)
Hydrocele of tunica vaginalis (n, (%))	1(0.77)	1(0.77)	1(0.78)	1(0.84)
Umbilical hernia (n, (%))	1(0.77)	1(0.77)	1(0.78)	2(1.68)

Note: n, number of observations.

## Discussion

The laparoscopic technique in PD catheter emplacement is gradually becoming preferred method for ESRD patients. Laparoscopic placement of PD catheter offers advantages such as minimal invasion, visualization of the peritoneal cavity, concomitant operation of omentectomy and adhesiolysis during the procedure^4^. Some reports have documented that this approach had benefits in decreasing operation time, lessening perioperative pain, and reducing catheter complications. However, most published studies are conducted in Occidental patients^8–10^. It is well known that the physical characteristics are different between Chinese and Occidental people. Chinese people are prone to have weaker abdominal muscles and less subcutaneous fat. Whether laparoscopic PD catheter insertion technique is suitable for Chinese patients and what are the catheter outcomes has not been well studied. Therefore, we collected and analyzed the data from 130 Chinese patients who underwent laparoscopic PD catheter emplacement since 2008. The overall results showed that the advanced laparoscopic PD catheter implantation was also suitable for Chinese patients and achieved satisfactory outcomes. As a retrospective study, 20% incidence of previous abdominal surgery in our study population reflected that our policy included the patients with prior abdominal operations. And the age range of the patients was between 15 years old and 83 years old, which reflected that our policy on all the patients was based on the principles of voluntary to accept laparoscopic surgery for PD catheter placement.

Our procedure design was similar to that in the Crabtree *et al*. series^8^. Compared with the procedure employed by Crabtree group, our technique was classified as advanced laparoscopy because the procedure included rectus sheath tunneling, prophylactic omentopexy, and adhesiolysis^11^. In addition, we also did some technical modifications: 1) the trocars were 3/5-mm (3-mm for patients with no need of adhesiolysis, 5-mm for those requiring adhesiolysis). In our series, we found that 3/5-mm trocars gave enough space for the manipulation accompanied with smaller incisions and less incidence of pericatheter leakage. 2) The trocar for catheter insertion was angled toward the symphysis pubis. Through this route, we could maximally avoid tissue and vessel injuries. 3) We didn’t place purse-string suture around the catheter at the anterior rectus sheath. In our experience, putting the deep cuff inner the rectus muscle could effectively prevent the catheter migration, so that would decrease catheter flow obstruction.

Nowadays, open PD catheter placement is the major method for PD catheter implantation in China. It was reported that catheter malfunction was 14.4% with 8.33% leakage by open approach in China^12^. We found that the catheter complications could be reduced by laparoscopic PD catheter placement in Chinese patients. Current study showed that the incidence of outflow obstruction (4.62%) and the pericatheter leakage(2.31%) in 130 cases was much less than the open insertion. And the occurrences of catheter outflow obstruction or pericatheter leakage were within three months after operation.

Consistent with other studies, the occurrence rate of outflow obstruction(4.62%) was within the range of other studies using laparoscopic surgery method for PD catheter implantation in Occidental (4–13%)^13^. Since omental wrapping was the major cause for outflow obstruction^14^, we also performed omentopexy during catheter implantation by laparoscopy. During the operation, the edges of the omentum were fixed onto the gastroepiploic peritoneum to prevent omental wrapping if the omentum was large or bulky.

It was reported that the incidence of leakage was 0–12.8% in patients received laparoscopic PD catheter placement^15, 16^. In our study, we observed pericatheter leakage in 2.31% patients (3 of 130), which was similar to the result of Crabtree *et al*.^8^. It is known that reducing the diameter of the trocars could prevent leakage. Here we used 3/5-mm trocar and didn’t place purse-string suture around the catheter at the anterior rectus sheath, showing a low rate of leakage. Moreover, our patients waited for 10 days to recover from the surgery before routine CAPD with standard 2 L dialysate, which might also contribute to the prevention of leakage.

Implantation of PD catheters using laparoscopic methods could help determine the presence and extent of intra-abdominal adhesions and direct the placement of catheters. It is strongly recommended in guidelines for laparoscopic PD catheter placement in those patients with prior abdominal surgery^17^. In our study, 26 out of 130 (20%) cases had previous abdominal surgeries, and 6 (23.08%) of them were noted to have intraperitoneal adhesions. In patients with no prior surgeries, only 5.77% patients (6 out of 104) displayed intraperitoneal adhesions. The rate of previous abdominal surgery is similar to the study by Chen *et al*.^18^. In their study, 14.8% had prior abdominal surgery in a total of 122 patients, while they did not mention adhesiolysis^18^. Wang *et al*. reported that 25% required adhesiolysis in a total of 20 patients who received laparoscopic catheter placement with prior abdominal operations^19^.

Crabtree *et al*. retrospectively studied 436 catheter placements and found that 80 out of 252 (31.8%) catheter placement procedures in patients with prior abdominal operations required adhesiolysis, while only 6(3.3%) of 184 procedures in patients without prior surgery needed adhesiolysis. Interestingly, the study showed that patients with minimally invasive procedures (e.g. uterine tube ligation and other laparoscopic interventions) frequently required adhesiolysis. However, patients with major abdominal procedures (such as aortic repair, colorectal resection, hysterectomy, and transabdominal nephrectomy) required no adhesiolysis during catheter placement^20^. In our study, we found that it was not possible to predict the occurrence of significant postsurgical adhesions based upon the type of previous surgical procedure, which is in agreement with Crabtree *et al*. Instead, the incidence of adhesion was significantly related to how many times of previous abdominal surgeries the patient had.

In our study, 12 out of 130 patients were found to have peritoneal adhesions. And 2 of them had catheter outflow dysfunction and 1 patient had pericatheter leakage. The incidence rates of outflow dysfunction and pericatheter leakage were lower than that reported by Ogunc^21^. A study by Keshvari *et al*. showed that there was no difference in the types of mechanical complications or revisional interventions following catheter insertion between the groups with and without intraperitoneal adhesions, and overall catheter survival was also not significantly different^22^.

In summary, in the current study, we reported the results and experience of PD catheter placement via advanced laparoscopy in 130 Chinese patients. The results indicated that this technique is also safe and effective in Chinese patients, especially for patients with prior abdominal surgeries. The laparoscopic operative complications were few and PD catheter function was satisfactory.

## Materials and Methods

### Study design

We performed a retrospective analysis of all patients who underwent laparoscopic PD catheter placement in our medical center from October 2008 to September 2016. In this study, all patients who were candidates for CAPD were diagnosed with end-stage renal disease at our dialysis center and consent to accept laparoscopic surgery for PD catheter placement. Patients were excluded if they had an active intraabdominal infection. Previous abdominal surgery was not an exclusion criterion. Patient characteristics such as age, gender, cause of ESRD, and history of prior abdominal surgery were collected. Perioperative details such as pre-existence of adhesion, status of omentum, operation time, performance of adhesiolysis, incidence of bleeding or perforation, and successful rate were collected. The catheter complications (dialysate leak, outflow obstruction, and hernia), and follow-up information including mortality and peritoneal dialysis outcomes. Follow-up time was from the day of PD catheter placement to March 2017 for patients remained on PD. Otherwise, the end point will be substituted by the date of patient’s death or the date of catheter’s permanent removal.

The study was approved by the Ethics Committee of Shunde Hospital of Southern Medical University. Written informed consent was obtained from all patients or family members before their participation of this study. All procedures were conducted in accordance with the approved guidelines which also conformed to the principles of the Helsinki Declaration.

### Laparoscopic PD catheter placement procedure

The PD catheter has two-cuff with a straight section (Tyco Healthcare Group LP, USA). The catheter is 41-cm long, and its diameter is 5-mm. The placement surgery was performed under a laparoscope (Sanofi Pasteur MSD). The instruments consist of 3-mm 0° laparoscopic tube, three puncture tubes whose diameters are 3-mm to 5-mm.

All patients were under intravenous anesthesia during the operation. The laparoscopic surgical technique is as follows: Total of three incisions were made. The first incision was under the navel (port A). The second incision was 2 to 5 cm lower than the navel and was on the right rectus muscle (port B). The third incision for 5 mm trocar was in the left lower quadrant (port C) which was parallel to Mcburney point as assistance. 1) At port A, pneumoperitoneum was established on 8–10 mmHg, and a 3-mm trocar was introduced into the peritoneal cavity, the peritoneum was evaluated by using a 0° laparoscope. 2) At the port B, a 3/5-mm trocar was introduced into the peritoneal cavity and advanced toward the symphysis pubis. This trocar orientation was very important. Adhesiolysis was performed if there was abdominal adhesion. If the omentum was large or bulky, the edges of the omentum would be fixed onto the gastroepiploic peritoneum. Then the catheter was inserted at port B through the rectus muscle and sheath directed to pubic symphysis. 3) At port C, through the trocar, a bowel clamp was introduced into the peritoneal cavity, and then clamped the end of the catheter to pelvic cavity. The catheter end naturally curved in the Douglas’ pouch. The deep cuff was inner rectus outside the peritoneum. We didn’t suture pouch of the anterior rectus sheath. 4) 500 ml of 0.9% sodium chloride were injected into the abdominal cavity to test the flow. 5) The distal end of the catheter was tunneled out leaving the second cuff in the subcutaneous tunnel. The A and C ports just need a stitch.

### Postoperative management

All patients having the operation were in hospital. The patients initialed 0.5-L dialysate in and out the cavity of 4 circulation everyday within 3 days after catheter placement. The volume was then gradually increased. After 3 to 5 days, the patient was converted to intermittent peritoneal dialysis. Finally, a standard 2-L dialysate of CAPD was performed at about 10 days.

### Statistical analysis

Continuous variables were presented as means ± standard deviations and categorical variables as frequencies with associated percentages. Probability distributions for catheter survival were estimated using the method of Kaplan and Meier. Statistical analyses were performed with the Statistical Package for Social Science ver. 16.00 (SPSS Inc, Chicago, IL, USA).

## Electronic supplementary material

Adhesiolysis during peritoneal dialysis catheter emplacement

Omentopexy during peritoneal dialysis catheter emplacement

Peritoneal dialysis catheter emplacement
